# Reply to Németh and Garvie: Evidence for lonsdaleite in ureilite meteorites

**DOI:** 10.1073/pnas.2305559120

**Published:** 2023-05-08

**Authors:** Andrew G. Tomkins, Nicholas C. Wilson, Colin MacRae, Alan Salek, Matthew Field, Helen E. A. Brand, Andrew D. Langendam, Natasha R. Stephen, Aaron Torpy, Zsanett Pintér, Lauren A. Jennings, Dougal McCulloch

**Affiliations:** ^a^School of Earth, Atmosphere and Environment, Monash University, Melbourne, VIC 3800, Australia; ^b^Commonwealth Scientific and Industrial Research Organisation Mineral Resources, Microbeam Laboratory, Melbourne, VIC 3169, Australia; ^c^Physics, School of Science, RMIT University, Melbourne, VIC 3001, Australia; ^d^Royal Melbourne Institute of Technology Microscopy and Microanalysis Facility, RMIT University, Melbourne, VIC 3001, Australia; ^e^Australian Synchrotron, Clayton, Melbourne, VIC 3168, Australia; ^f^Plymouth Electron Microscopy Centre, University of Plymouth, Plymouth, Devon PL4 8AA, United Kingdom

In a recent letter to the editor, Nemeth and Garvie (1) questioned our experimental evidence for lonsdaleite in ureilite meteorites, which we suggested formed via in situ chemical fluid/vapor deposition (2). The hexagonal form of diamond, known as lonsdaleite, was first reported in the Canyon Diablo meteorite where it likely formed from graphite via high shock pressures (3). Nemeth and Garvie previously argued that the lonsdaleite in Canyon Diablo is instead a defective diamond (4) or combined graphite/diamond complexes coined, “diaphites” (5).

Their letter (1) suggested that within experimental error, our electron diffraction results (2) can be attributed to graphite, cubic diamond, or diaphites. In our work, polycrystalline Pt was used as a standard to ensure high calibration accuracy, better than 1%, for diffraction analysis. At this level of accuracy, it is easy to distinguish between key phases, including the hexagonal set of {110}/{100} diffraction spots from lonsdaleite 2.18 Å and graphite 2.23 Å (>2% difference) when viewed down <001> (*SI Appendix*, Fig. S5). The nearest diffraction spots from diamond [the {111} at 2.06 Å] can also be ruled out. Multiple diffraction patterns taken at different orientations from the same crystal (*SI Appendix*, Fig. S6) only match lonsdaleite.

Electron energy loss spectroscopy (EELS), particularly in combination with the diffraction analysis, provides conclusive evidence for lonsdaleite. In addition to detailed information on local bonding arrangements, EELS can measure density from the plasmon peak position (6). In carbon solids, this approach has been verified both experimentally and theoretically (7). We determined the density of our lonsdaleite crystals to be 3.5 g/cm^3^ (*SI Appendix*, Fig. S5*E*), which rules out the possibility that the <001> diffraction patterns (*SI Appendix*, Fig. S5*B*) were collected from graphite (much lower density of 2.26 g/cm^3^); the hexagonal symmetry is not compatible with diamond. This density also excludes the possibility that this region contained significant quantities of diaphite (expected density ~2.9 g/cm^3^). Recent hardness measurements (8) also show that the lonsdaleite in our samples has extreme hardness, not consistent with graphite or diaphite.

Our interpretation of cathode luminescence (CL) measurements was also questioned; they noted that N-doped diamonds have a broad luminescence band at 535 nm (9). This peak has a full-width half maximum (FWHM) of 450 meV, whereas the peak we observe (*SI Appendix*, Fig. S3) is much sharper (FWHM of 27 meV) and is accompanied by a set of phonon replicates with approximately 130 meV spacing. This indicates that the lonsdaleite we observed and N-doped diamond have different defect structures.

Lastly, the fast Fourier transform shown in *SI Appendix*, Fig. S8 matches the {100}/{110} reflections of lonsdaleite. This figure also shows the well-known orientation relationship between the graphite <001> and lonsdaleite <120> directions (4), which is consistent with the theoretical transformation pathways between graphite and lonsdaleite (10).

We suggest here that the diaphite described by Nemeth and Garvie in Canyon Diablo (4, 5) may be the precursor to lonsdaleite formation in ureilite meteorites. Intense shock is needed to form diaphite, and in the ureilites, intense shock immediately preceded the chemical vapor/fluid deposition process. Shock may have created the out-of-equilibrium structure that facilitated replacement by lonsdaleite.

## References

[1] P. Nemeth, L. A. J. Garvie, Questionable lonsdaleite identification in ureilite meteorites. Proc. Natl. Acad. Sci. U.S.A. **120**, e2304890120 (2023). 37155887 10.1073/pnas.2304890120 PMC10193955

[2] A. G. Tomkins , Sequential lonsdaleite to diamond formation in ureilite meteorites via chemical fluid/vapor deposition. Proc. Natl. Acad. Sci. U.S.A. **119**, e2208814119 (2022). 36095186 10.1073/pnas.2208814119 PMC9499504

[3] C. Frondel, U. B. Marvin, Lonsdaleite, a hexagonal polymorph of diamond. Nature **214**, 587–589 (1967).

[4] P. Nemeth , Lonsdaleite is faulted and twinned cubic diamond and does not exist as a discrete, material. Nat. Commun. **5**, 5447 (2014). 25410324 10.1038/ncomms6447

[5] P. Nemeth , Shock-formed carbon materials with intergrown sp^3^- and sp^2^-bonded, nanostructured units. Proc. Natl. Acad. Sci. U.S.A. **119**, e2203672119 (2022). 35867827 10.1073/pnas.2203672119 PMC9335297

[6] R. F. Egerton, Electron Energy-Loss Spectroscopy in the Electron Microscope (Springer Science & Business Media, 2011).

[7] A. C. Ferrari , Density, sp^3^ fraction, and cross-sectional structure of amorphous carbon films determined by x-ray reflectivity and electron energy-loss spectroscopy. Phys. Rev. B **62**, 11089 (2000).

[8] X. Huang , Hardness of nano- and microcrystalline lonsdaleite. Appl. Phys. Lett. **122**, 081902 (2023).

[9] A. M. Zaitsev, K. S. Moe, W. Wang, Defect transformations in nitrogen-doped CVD diamond during irradiation and annealing. Diam. Relat. Mater. **88**, 237–255 (2018).

[10] S. Scandolo, M. Bernasconi, G. L. Chiarotti, P. Focher, E. Tosatti, Pressure-induced transformation path of graphite to diamond. Phys. Rev. Lett. **74**, 4015 (1995). 10058391 10.1103/PhysRevLett.74.4015

